# Pancreatic draining lymph nodes (PLNs) serve as a pathogenic hub contributing to the development of type 1 diabetes

**DOI:** 10.1186/s13578-023-01110-7

**Published:** 2023-08-28

**Authors:** Fei Sun, Chun-Liang Yang, Fa-Xi Wang, Shan-Jie Rong, Jia-Hui Luo, Wan-Ying Lu, Tian-Tian Yue, Cong-Yi Wang, Shi-Wei Liu

**Affiliations:** 1 Shanxi Bethune Hospital, Shanxi Academy of Medical Science, Tongji Shanxi Hospital, Third Hospital of Shanxi Medical University, Taiyuan, China; 2 NHC Key Laboratory of Respiratory Diseases, Department of Respiratory and Critical Care Medicine, The Center for Biomedical Research, Tongji Hospital, Tongji Medical College, Huazhong University of Science and Technology, Wuhan, China; 3 Devision of Nutrition, Tongji Hospital, Tongji Medical College, Huazhong University of Science and Technology, Wuhan, China

**Keywords:** Type 1 diabetes (T1D), Pancreatic draining lymph nodes (PLNs), Signal inputs, PLN remodeling, Signal outputs

## Abstract

Type 1 diabetes (T1D) is a chronic, progressive autoinflammatory disorder resulting from the breakdown of self-tolerance and unrestrained β cell-reactive immune response. Activation of immune cells is initiated in islet and amplified in lymphoid tissues, especially those pancreatic draining lymph nodes (PLNs). The knowledge of PLNs as the hub of aberrant immune response is continuously being replenished and renewed. Here we provide a PLN-centered view of T1D pathogenesis and emphasize that PLNs integrate signal inputs from the pancreas, gut, viral infection or peripheral circulation, undergo immune remodeling within the local microenvironment and export effector cell components into pancreas to affect T1D progression. In accordance, we suggest that T1D intervention can be implemented by three major ways: cutting off the signal inputs into PLNs (reduce inflammatory β cell damage, enhance gut integrity and control pathogenic viral infections), modulating the immune activation status of PLNs and blocking the outputs of PLNs towards pancreatic islets. Given the dynamic and complex nature of T1D etiology, the corresponding intervention strategy is thus required to be comprehensive to ensure optimal therapeutic efficacy.

## Introduction

As a prototypical autoimmune disease, type 1 diabetes (T1D) stems from the breakdown of self-tolerance and subsequent relentless immune attack which destroys pancreatic islet β cells, thereby leading to insulin deficiency [1–5]. The etiologies underlying T1D are yet to be fully addressed, but are associated with genetic predisposition, epigenetic reprogramming and environmental cues including diet, lifestyle change, microbiota alteration and infection of specific viral strains [6–9]. All these intrinsic abnormalities and extrinsic insults are deemed to initiate islet autoreactive immune responses. Damage associated molecular patterns (DAMPs) along with autoantigens released from dying β cells are the major drivers of autoimmune priming [10, 11]. On the other hand, disrupted integrity of the intestinal barrier allows translocation of microbial components to the remote area, which then act as immunostimulatory adjuvants to exacerbate β cell destruction [12, 13]. In particular, viral infections could directly interfere with β cell function, but the deteriorative effect largely comes from infection-induced interferonopathy, a “spillover effect” of the anti-viral response [14, 15].

Single-cell techniques applied in peripheral blood, pancreatic draining lymph nodes (PLNs) and pancreas have greatly advanced our understanding of cell components involved in T1D development [16]. Strikingly, single-cell RNA sequencing (scRNA-seq) with 4-week, 8-week and 15-week old non-obese diabetic (NOD) mice found that the immune infiltration is already identifiable as early as 4-week of age, rapidly progresses at around 8-week old and peaks at 15-week old [17]. T1D is therefore acknowledged as a chronic progressive inflammatory disorder. In this case, the breakdown of immune tolerance is a gradually occurring process, but most critically, our body could exploit versatile approaches to counterbalance the overactive autoimmune responses to protect the residual β cell mass. ScRNA-seq of human pancreas revealed the unexpected immune regulatory function of ductal epithelial cells [18, 19], and upon IFN-γ stimulation, β cells actively upregulate PD-L1 expression to resist autoinflammatory assault [20]. Additional immunological self-limiting mechanisms are also found including regulatory T cell (Treg) adaptation, activation-induced cell death (AICD) and the exhaustion of effector T cells (Teff), which collectively put a brake on the derailed immune responses [21, 22].

Given that T1D is resulted from autoimmune destruction of islet β cells, the crosstalk between β cells and islet resident immune cells plays an initiative part and determines the tissue specificity of T1D, but the destructive autoimmune response is owing to the signaling amplified in organized lymphoid structures. Mounting studies have demonstrated the presence of tertiary lymphoid organs (TLOs) in the peri-islet milieu [23]. TLOs are formed in response to lymphotoxin signaling, and therefore, the removal of PLNs in NOD mice cannot entirely prevent T1D development [17, 24, 25]. In general, TLOs are normally visible at 14–20 weeks of age in NOD mice [24, 26]. As a result, it is very unlikely that TLOs could replace the role of PLNs in T1D pathogenesis, especially at the early stage of disease development. Indeed, excision of PLNs at 3 weeks almost completely protects NOD mice from insulitis and diabetes, but the goal cannot be achieved once it is conducted at 10 weeks of age [17]. The knowledge of PLNs as a place of aberrant immune response is continuously being replenished and renewed, and what we presented here aims to piece together those valuable up-to-date findings, and to delineate the comprehensive landscape of T1D pathogenesis from a PLN-centered perspective.

## PLNs integrate priming signals from diverse sources of input

Pancreas is the primary source of input signals for efficient triggering of immunological events within PLNs. Recurrent exposure to islet-specific antigens is deemed to contribute to the early initiation of T1D [27]. Pancreatic islet β cell-derived granules containing catabolized insulin peptide fragments (e.g., insulin B:12-20) are released into circulation or near the neighborhood, taken up, and presented by antigen-presenting cells (APCs), which ultimately enhances CD4^+^ T cell diabetogenicity in various lymphoid tissues, especially PLNs, as evidenced by the presence of insulin specific germinal centers (GCs) [28]. Dendritic cells (DCs) serve as a bridge linking β cell damage to the activation of adaptive immune system [29]. Defects in NOD DCs has been ascribed to the *Idd10/17/18* region, which hinders the generation of tolerogenic DCs and arrests DCs in a maturing phase, thereby producing more IL-12 but less IL-10 [30]. Prior to overt lymphocytic insulitis, CD8a^+^ DCs accumulate at the edge of islet. The frequency of CD8a^+^ DCs reduces in the pre-diabetic pancreas rather than in the PLNs, and the expression of tolerogenic markers such as CCR5, CLEC9A, and IL-10, is down-regulated. These data indicate that alteration of DC state and loss of peri-islet tolerance might precede the breakdown of tolerance in PLNs [31]. There are two major subsets of islet resident DCs: CD103^+^ DCs derived from pre-DCs, and CD11b^+^ DCs originated from circulating monocytes. CD103^+^ DCs are adept at cross-presenting islet autoantigens by migrating towards PLNs, while CD11b^+^ DCs are more phagocytic and preferentially stay in the islet [32]. Physiological β cell death, occurring around 2 weeks of age in all mouse strains, goes awry in NOD mice, which provides primordial diabetogenic antigen to CD11b^+^ DCs and provokes T cell activation in PLNs [33]. In contrast, BATF3-dependent CD103^+^ DCs make up a minor population of islet APCs in newborn NOD mice; however, by 4 weeks of age, the proportion of CD103^+^ DCs surges in concomitant with the accession of T cells into islets. Ablation of BATF3 results in a lack of CD103^+^ DCs in both pancreas and PLNs, thereby preventing autoreactive T cell activation and T1D development [34]. An amplification loop is also identified between T cells and DCs, as islet infiltrating T cells are able to further upregulate the expression of CD40, CD80 and CCR7 on DC surface, which augments their potency to prime more autoreactive T cells in PLNs [35]. In addition to DCs, B cells partially contribute to T1D pathogenesis by immunoglobulin (Ig)-mediated antigen capture and the priming effect on diabetogenic T cell response [36]. Therefore, although the pathogenic role of B cell-secreted autoantibodies (Ab) is an issue under debate, autoreactive B cells may act as APCs necessary for the initial activation of β cell reactive CD4 T cells [37].

Notably, intra-islet APCs capture antigenic peptides, get matured and obtain the migratory capacity towards PLNs via the afferent lymphatic vessels [38]. Lymph-angiogenesis represents a pathological feature commonly observed in chronic inflammatory disorders, particularly in the case of insulitis in T1D setting. Vascular endothelial growth factors receptor 3 (VEGFR3) is critically involved in the above process, and VEGFR3 blockade reduces multiple low dose streptozotocin (MLDS)-induced immune responses in PLNs [39]. By injecting indocyanine green (ICG) into parenchyma in the anterior or posterior surface of the pancreas head, seven main pancreatic lymphatic drainage pathways were identified [40]. A similar technique may be applied to NOD mice to reveal the lymphatic draining pathways, given lymphatic system is tightly associated with the initiation or resolution of pancreatitis [41]. Unfortunately, relevant studies on whether targeting lymph-angiogenesis is a feasible approach for T1D treatment are lacking thus far.

Specific strains of viral infections also contribute to the motivation of PLNs and the priming of autoinflammatory reactions. Orally infected rhesus monkey rotavirus (RRV) makes its presence in PLNs by extra-intestinal spread, which activates regional APCs and elicits a Th1 biased adaptive immune response. Rotavirus infection in at-risk children positively correlates with T1D progression and accelerates T1D onset in a mouse model [42]. Mechanistically, rotavirus infection of NOD mice enhances the expression of MHC-I molecule on PLN B cells and promotes the proliferation of autoreactive T cells possibly through bystander activation [43]. In rats, Kilham rat virus (KRV) infection reproducibly induces acute T1D in genetically predisposed BB/Wor strain. By in situ hybridization, the tissue tropism of KRV infection was unraveled. Interestingly, KRV mRNA and DNA were readily detected in peripancreatic lymphoid tissues while were hardly seen in the pancreas following 5 days of infection [44]. Consistently, the T1D-inducing effect of KRV infection is attributed to B cell and plasmacytoid DC (pDC) activation in PLNs. Microarray analysis revealed that the upregulated genes elicited by KRV infection were predominantly IFN-γ-induced chemokines and genes associated with IL-1 pathways, interferon production, and downstream signaling molecules [45]. On the contrary, certain viral inputs may alleviate the progression of T1D. For instance, intraperitoneal or intranasal infection of murine gammaherpesvirus-68 (MHV-68) delays T1D onset by reducing dendritic cell antigen presentation and rendering PLN autoreactive T cells at a naïve state [46]. Therefore, the immune regulatory role of viral infections may vary under the context of T1D.

Gut-derived signals are another important source of input that affects the immune status of PLNs. From the perspective of development, a preferential trafficking route exists from the gut to PLNs, and PLN resident T cells can also be activated by antigens drained from the peritoneum and the gastrointestinal tract [47]. An appealing hypothesis proposed that dietary intake of wheat gluten triggers T1D pathogenesis by releasing dipeptidyl peptidase IV (DPP4)-cleaved X-pro peptides. Gluten-derived peptides would be ingested by intestinal DCs, which are then recruited into PLNs by chemokines CCL19/CCL21 to activate β cell reactive lymphocytes [48]. A similar antigen mimicry approach is adopted by pathogenic gut microbiome. The *hprt4-18* peptide derived from the human gut commensal *Parabacteroides distasonis* activates T cell clones of T1D patients that are specifically directed at an epitope in the B-chain of insulin (insB:9-23), and as a result, the seroconversion rates are consistently higher in children whose microbiome harbors sequences capable of producing the *hprt4-18* peptide [49]. Moreover, the dysbiosis of gut microbiota, disruption of the intestinal barrier integrity and microbial translocation are construed as the key pathological events in T1D pathogenesis as well [50, 51]. For example, streptozotocin (STZ) treatment would cause a “leaky gut” permitting the translocation of microbial products into PLNs, where they are probed by the nucleotide-binding oligomerization domain containing 2 (NOD2), inducing pathogenic Th1 and Th17 response [52]. Additionally, functional and metabolic alterations of gut microbiome, featured by the decreased butyrate production and bile acid metabolism along with increased lipopolysaccharide biosynthesis, are observed in T1D children [53]. Particularly, the combination of 18 bacterial species and fecal metabolites provides prognostic value for T1D [53], which lays the foundation for microbiota-based T1D therapies including fecal microbiota transfer (FMT) [54] and the supplementation of beneficial bacterial species [55]. Taken together, although gut-associated lymph nodes are sources of intermediate diabetogenic lymphocytes, they are likely engaged in the early phase of T1D initiation [56].

**Fig. 1:**
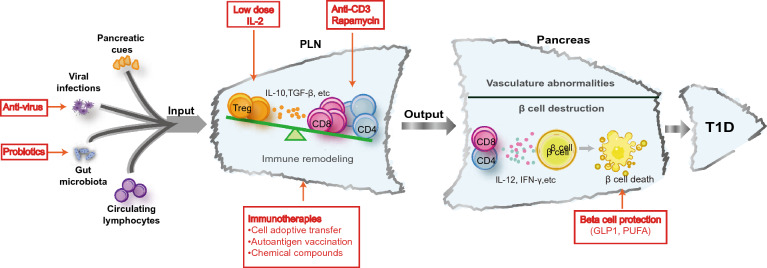
Pancreatic draining lymph nodes (PLNs) integrate signal inputs from various sources and undergo substantial immune remodeling to elicit anti-islet response. Pancreas derived autoantigens (soluble or presented by APCs), viral infections, gut microbiota components and circulating lymphocytes get access into PLNs, leading to and/or boosting the imbalance of Treg/Teff. The primed autoreactive T cells then serve as the major PLN outputs that infiltrate the pancreas and are responsible for T1D initiation. *APC* antigen-presenting cell, *Treg* regulatory T cell, *Teff* effector T cell

PLNs constantly exchange cell components with the peripheral blood and circulating diabetogenic T cells tend to choose PLNs as the priority to habitat. Circulating B cells access into PLNs mainly by their surface expression of mucosal addressin cell adhesion molecule 1 (MAdCAM-1) and α4β7 integrin, and partly by the presence of L-selectin or LFA-1. Upon their arrival in PLNs, they sense, capture and present the drained autoantigens to T cells [57]. Using a T1D adoptive transfer model in NOD mice, by analysis of the transferred T cells in the pancreas and lymphoid organs including thymus, spleen, and lymph nodes from pancreatic, mesenteric, axillary, inguinal and combo-aortic areas, it was interestingly discovered that the transferred T cells are readily and predominantly infiltrated into PLNs, where they undergo the process of activation and acquisition of diabetogenicity [58]. This phenomenon is corroborated by the adoptive transfer of antigen-specific BDC2.5T cells. Before insulitis is detectable, the transferred T cells are found to only proliferate in PLNs, indicating that β cell-derived antigens are similarly and predominantly transported into PLNs, although small amounts of antigens could be spread into remote areas [59]. Altogether, PLNs receive signals from the pancreas, gut, viral infection, and circulation (Fig. 1). These diverse external inputs are integrated in PLNs and finally transformed into abnormal islet autoreactive immune responses, which would be discussed in the following sections.

## PLN remodeling is featured by the perturbation of immune microenvironment

In recent-onset T1D patients, histological examination revealed decreased primary B cell follicle frequency, fewer follicular dendritic cell (FDC, CD21, and CD35 positive) networks, and accumulation of extracellular matrix glycosaminoglycan hyaluronan (HA) and HA binding proteins in PLNs [60, 61]. By classifying 5-week old NOD mice into insulin autoantibody (IAA^+^) group and IAA^−^ group along with comparative analysis, it was found that the differentially expressed genes (DEGs) are enriched in tissue reconfiguration and Th1 immunity, suggesting an early immunological rewiring in PLNs [62]. PLN remodeling is accompanied by a shift from immune tolerance to the state of immune activation. Breakdown of self-tolerance is a prerequisite for the autoreactive response, and anomalies in tolerizing mechanisms take the principal responsibility.

Generally, PLN remodeling is characterized by the alteration of stromal cells. Fibroblastic reticular cells (FRCs) form the scaffold to support the PLN architecture and physiologically present self-antigens to induce T cell tolerance. FRC networks in NOD PLNs display larger reticular pores than non-obese diabetes-resistant (NOR) controls, and thus engage with more T cells, which possibly serves as a compensatory anti-inflammatory mechanism [63]. Lymph node stromal cells (LNSCs) are also physiologically involved in T cell tolerance induction in human T1D patients, and similarly, NOD mice PLN-derived LNSCs display enhanced tolerogenic phenotype along with increased antigen-presenting potential to offset DC-induced T cell activation [64]. Deformed epidermal autoregulatory factor 1 (DEAF1) is a transcription regulator that promotes the expression of peripheral tissue antigens (PTA) in LNSCs. As forward of T1D progression, the alternatively spliced dominant-negative isoform DEAF1-Var1 is upregulated in PLNs (through splicing factor Srsf10 and Ptbp2), which reduces PTA expression and possibly promotes the loss of peripheral tolerance [65]. Reduction of DEAF1 function downregulates the expression of eukaryotic translation initiation factor 4 gamma 3 (Eif4g3), which modulates the translation of various genes involved in PTA presentation (such as aminopeptidase N), as revealed by the polysome profiling [66]. The expression of tissue-specific antigens (TSAs) mediated by the autoimmune regulator (AIRE) in the thymus is essential for central tolerance induction, while DEAF1 may serve as a master regulator manipulating the expression of PTAs and peripheral tolerance induction [67, 68]. Therefore, PTA-mediated peripheral tolerance induction plays an instructive role in T1D initiation [69].

It is worthy of note that PLN resident and immigratory APCs are decisive for the ultimate tolerance breakdown and priming of autoimmune reactions. Compared to DCs isolated from PLNs of control mice or axillary/inguinal (A/I) LN of NOD mice, DCs from NOD PLNs form larger clusters with T cells (increase with age) which comprise a major source of proliferating T cells. The cluster formation is specific, as NOD PLN DCs fail to cluster with A/I T cells and in turn A/I DCs fail to cluster with PLN T cells [70]. The DNAX-activating protein of 12 kDa (DAP12) is an adaptor molecule expressed on lymphoid and myeloid cells. DAP12 in DCs facilitates the activation of PLN Treg cells and serves as a tolerance mechanism to β cell-derived antigens. *DAP12* deficiency in BDC2.5/B6g7 TCR transgenic mice manifests higher activation of PLN T cells and more rapid T1D onset, implying the critical role of DC in dictating the direction of tolerance or immunity of PLNs [71]. B cells play an elusive part in T1D pathogenesis regarding the production of autoantibodies [72]. Eight-hundred sixty-three human IgG antibodies were cloned from 4092 single B cells from PLNs and peripheral blood. Surprisingly, only 2 clones showed reactivity to insulinoma-associated antigen 2 (IA-2), while the rest of them were negative for commonly known autoantigens including IA-2, GAD65 and zinc transporter 8 (ZnT8), indicating an infrequent presence of autoantigen-specific IgG^+^ B lymphocytes in PLNs from IAA-positive individuals [73]. Marginal zone B (MZB) cells are detected in almost 80% of NOD mice by 16-week old and the population expands along with T1D progression. These MZB cells are hyperresponsive to TLR, CD40 and S1P, and express MHC-II, CD80 and CD86, by which they serve as potent APCs to prime diabetogenic T cells within PLNs [74]. Therefore, B cells would probably assist DCs in the transition of PLN state from tolerance to immunity.

The breakdown of self-tolerance is followed by unrestrained autoreactive T cell response, which contributes to the long-lasting and unresolved T1D progression [75, 76]. PLN memory CD4^+^ T cells and pancreatic memory CD4^+^ T cells share restricted TCRβ usage, and the majority of public clonotypes express TRBV13-2 (Vβ8.2) gene segment. Further analysis of CDR3β sequences revealed rare clones of well-identified diabetes-related clonotypes, including those recognizing IGRP, insulin B:9–23 and chromogranin, which reflects the potential occurrence of intra- or inter-molecular epitope spreading and the hypermutation nature of TCR [77]. A high degree of clonal expansion was observed in PLNs from long-term diabetic patients [78]. However, despite the promiscuous TCR clones within PLNs, the disease-causing clonotypes may be limited [79]. In NOD mice, T cells specifically recognizing HIP2.5 epitope (a fusion of insulin C-peptide and chromogranin A fragment) account for around 40% of islet-infiltrating T cells at both prediabetic and diabetic stages [80]. In humans, GAD65 reactive TCR is present in 38.9% of examined patients, which contributes > 25% reactive TCRβ (TRB) within the conventional T cells isolated from PLNs [81].

Crosstalk between APCs and T cells is indispensable for efficient T cell priming. NOD mice harbor a unique MHC-II genotype (I-Ag7), which presents β cell-derived naturally processed peptides mainly coming from proteins associated with neuronal or neuro-endocrine cell types (e.g. synaptotagmin, neuromodulin, and amyloid β) or proteins associated with secretory granules (e.g. secretogranin and chromogranin) to CD4^+^ T cells [82]. Replacement of I-Ag7 by I-E on DCs of NOD mice promotes the differentiation of autoreactive CD4^+^ T cells into antidiabetogenic autoregulatory T cells and protects against T1D progression [83]. Among different effector CD4^+^ T cell (Teff) subsets, Th1 is the most pathogenic one. Adoptive transfer of Th1 cells from BDC2.5 transgenic mice induces T1D in NOD/SCID mice. However, the transferred Th17 cells readily upregulate T-bet and secret IFN-γ upon exposure to IL-12, and neutralization of IFN-γ instead of IL-17 prevents T1D induced by the transfer of purified Th17 cells [84]. On the other hand, priming of diabetogenic CD8^+^ T cells requires the cross-presentation activity of DCs. Cross-presentation of islet antigens is inactive during neonatal life and gradually available when the inflammatory response becomes obvious [85]. NOD BMDCs pulsed with freeze-thawed insulinoma cells activate diabetogenic CD8^+^ T cells in the presence of TLR9 agonist and anti-CD40. Specifically, TLR9 affects the function of pDCs in PLNs, which produce type 1 interferons to participate in CD8^+^ T cell activation [86]. Notably, adoptive transfer of autoreactive CD8^+^ T cells alone results in clonal deletion in draining lymph nodes [87, 88], while co-delivery of autoreactive CD4^+^ T cells is required to provide essential help for the optimal activation of CD8^+^ T cells [89].

Immune regulatory cells are also present in PLNs to serve as a homeostatic mechanism to put a brake on the overactive immune response. Teff cells play a double-faceted role in T1D development. Teff cells other than induce islet destruction, they also boost Treg cell expansion to enhance their suppressive function in PLNs [90]. The number of PLN Tregs dramatically drops in NOD mice due to the impaired retention caused by the downregulation of SDF-1/CXCR4 axis [91]. Similarly, the frequency of T follicular regulatory (Tfr) cells, a specialized regulatory counterpart of T follicular helper (Tfh) cells, is reduced in PLNs of T1D patients, and re-supplementation of Tfr cells delays T1D development in mice [92]. The unbalanced immune status of human T1D is featured by functional defects in CD4^+^CD25^+^ Tregs in PLNs but not in peripheral blood [93, 94]. PLN Tregs inhibit in situ differentiation of islet-reactive CD8^+^ T cells, and the suppression is mediated by the TGF-β/TGF-βRII axis, as Treg cells could not control naïve or activated islet-reactive CD8^+^ T cells bearing a dominant-negative TGF-βRII genotype following adoptive transfer [95]. Likewise, a study argued that Treg function is not compromised during T1D initiation, rather conventional T cells showed reduced susceptibility to Treg-mediated suppression [96]. Such resistance of Teff cells to Tregs is mediated by the elevated IL-21 levels in PLNs, which probably contributes to the enhanced DC migratory capacity [97]. In addition to Treg cells, other regulatory cells are also involved in the modulation of PLN immune activation state. For instance, mice deficient in mast cells are more prone to multiple low dose STZ-induced insulitis, and adoptive transfer of mast cells confers resistance to T1D by promoting Treg cells and suppressing Th17 cells in PLNs [98]. NKT cells activated by alpha-galactosyl ceramide (alpha-GalCer) could induce the maturation of disease-protective DCs, which tolerizes pathogenic T cells in the PLNs. As a result, alpha-GalCer pretreatment reduces T1D incidence in mice [99, 100].

**Fig. 2:**
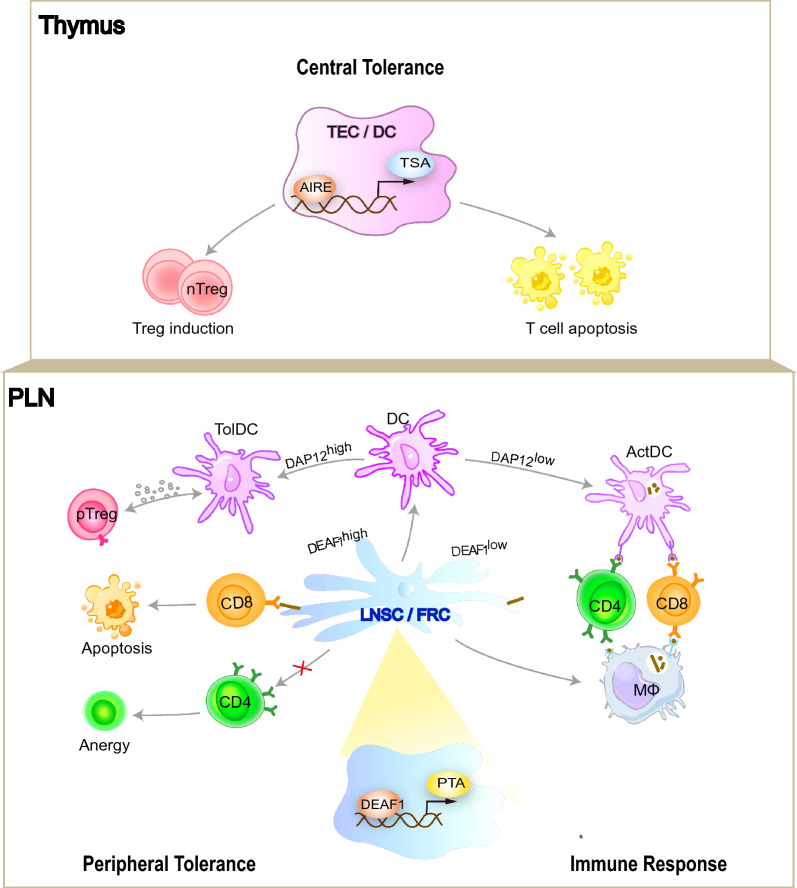
Breakdown of self-tolerance mechanism underpins T1D etiology. Briefly, self-tolerance is achieved at two different but related levels. Within the thymus, AIRE promotes the ectopic expression of tissue-specific antigens (TSAs) in thymic epithelial cells (TECs) and together with the presence of thymus resident DC, central tolerance is established through depleting autoreactive T cells (negative selection) and the induction of antigen-specific Treg cells. In parallel, within PLN, DEAF1 drives the ectopic expression of peripheral tissue antigens (PTAs) in lymph node stromal cells (LNSCs)/fibroblastic reticular cells (FRCs) and together with DAP12^hi^ DCs, peripheral tolerance is established to further solidify immune homeostasis. Abnormalities in organismal tolerizing mechanism is thus fundamental to the pathogenesis of autoimmune disorders including T1D. *Tol-DC* tolerogenic dendritic cell, *Act-DC* activated dendritic cell, *DAP12* DNAX-activating protein of 12 kDa, *DEAF1* deformed epidermal autoregulatory factor 1

In brief, intrinsic defects along with external inputs synergistically contribute to tolerance breakdown and immune activation in PLNs. The co-existence of both effector and regulatory mechanisms suggests that T1D pathogenesis is an outcome of immune imbalance gambled by the promiscuous immunological events, which explain the chronic and relapsing nature of the disease (Fig. 2).

## Autoreactive lymphocytes are exported from PLNs and infiltrate into the islet

After remodeling, PLNs become a formidable “military base” to store arsenal of weapons for β cell killing. Translocation of lymphocytes from PLNs to pancreatic islets (consisting of PLN egress, lymphocyte trafficking and islet infiltration) is crucial for T1D initiation. The BDC-Idd9 mice harbor BDC2.5 TCR transgenic T cells containing the *Idd9* genomic region originated from diabetes-resistant B10 mice. Unlike BDC T cells that predominantly accumulate in PLNs and pancreas, BDC-Idd9 T cells gather in splenic periarteriolar lymphatic sheaths, but both of them are comparable in terms of development, functional activation and proliferation [101]. Similarly, the NOD-Idd22 mice carry the diabetes-resistant ALR strain-derived *Idd22* genomic region (Chromosome8: D8Mit293-D8Mit137). This ALR-derived *Idd22* locus does not affect immune cell diabetogenicity, β cell resistance to cytotoxicity or proliferation of transferred CTLs in PLNs. However, β cell autoreactive T cells accumulate less in pancreatic islets due to the lower adhesion molecule expression on vascular endothelial cells and the consequent weaker adherence of T cells [102]. Vasculature abnormalities are indeed essentially implicated in T1D pathogenesis. Through contrast-enhanced ultrasound measurement, researchers found that islet microvasculature reorganization and blood flow dynamics precede T1D onset in various pre-clinical models, and islets have a denser microvasculature during diabetes progression [103]. Comparative microarray analysis revealed that genes involved in angiogenesis are specifically activated in NOD islets of 2–4 weeks of age [104]. In particularly, VEGFR2 is upregulated in inflamed islets and, as a result, inhibition of VEGFR2 ameliorates T1D progression, which supports that VEGFR2 is likely responsible for the enhanced vascularity and lymphocyte infiltration [105].

Adhesion molecules and chemokine–chemokine receptors, which are present on activated PLN-derived lymphocytes, are indispensable for the development of lymphocytic insulitis [106]. Mucosal addressin cell adhesion molecule-1 (MAdCAM-1) is expressed on islet vessels of NOD mice early during lymphocyte accumulation in islets. Integrin α4β7^hi^ T cells in NOD mice are mainly come from PLNs or spleen, rather than mucosal lymphoid tissue, which infiltrate into islet through binding to MAdCAM-1 [107]. Alternatively, high endothelial venules (HEVs) in inflamed islets co-express CCL21 and CCL19, which recruit CCR7^+^ T cells from bloodstream into islets. Blockade of CCR7 abolishes 70% of T cell infiltration while not affecting B cells [108]. Intravital two-photon imaging demonstrated that peri-vascular CD11c^+^ cells govern T cell extravasation by secreting plentiful and redundant chemokines. For this reason, depletion of peri-vascular CD11c^+^ cells, instead of blocking limited chemokine–chemokine receptor signaling pathways, is more efficient in preventing the entrance of lymphocytes into islets [109]. Intriguingly, activated T cells could upregulate the expression of insulin receptors (IRs). IR positivity not only helps sense insulin for enhanced metabolic activity but also serves as an atypical chemokine receptor that directs the migration of T cells towards islets following the concentration gradient of insulin [110].

**Fig. 3:**
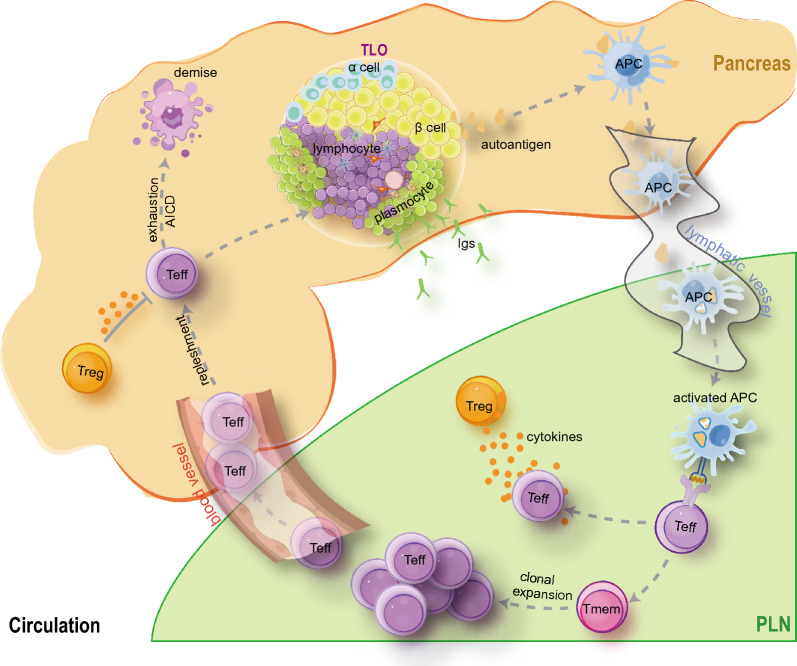
Pancreatic draining lymph nodes (PLNs) orchestrate and perpetuate the vicious cycle of islet-specific autoimmune reaction. Effector T cells (Teff) are efficient in β cell killing and are organized into specialized tertiary lymphoid organs (TLOs) with chronic T1D progression. Autoantibodies (Igs) produced by plasmocytes act as the early immune biomarker of T1D initiation and the autoantigens generated from dead islet β cells are presented by antigen presenting cells (APCs) to prime autoreactive T cells in PLNs. A minor population of autoreactive T cells are present in the form of stem like memory T cells (Tmem) to continuously fuel anti-islet immunity, considering that Teff have a short lifespan and would not persist once arriving at the islet niche

The stepwise, continuous spectrum of immune cell infiltration is best exemplified by CD8^+^ T cells, which experience distinct states of naïve, effector, memory, stem-like memory, or exhaustion. After leaving PLNs and arriving at islets, CD8^+^ T cells gradually gain higher expression of the cytotoxic effector markers, granzyme B, IFN-γ, and CD107a [111]. Activated CD8^+^ T cells face up with the fate of either becoming exhausted or dead after killing [21, 112]. TCF1^hi^ stem-like memory CD8^+^ T cells are a minor but unique cell population that possesses the characteristics of both memory cells and stem cells [113]. They reside in PLN and provide a persistent output of autoreactive CD8^+^ T cells that enter the islet and replenish the depleted mission-completed ones [113, 114]. The presence of TCF1^hi^ stem-like CD8^+^ T cells is also confirmed in conditions like tumors, and cDC1 is required for their maintenance [115]. Therefore, it is not surprising to observe decreased PLN cellularity and T cell number in NOD mice after disease onset [116], and the turnover of autoreactive lymphocytes may contribute to the remission-relapsing phases of T1D progression (Fig. 3).

As part of the compensatory protective mechanism, Treg cells also migrate from PLNs to the pancreas. In response to IFN-γ produced by Teff cells, antigen-specific ICOS^+^ Treg cells preferentially express CXCR3 in PLNs and are chemoattracted by CXCL9, CXCL10, and CXCL11 derived from intra-pancreatic APC populations and β cells, serving as a homeostatic mechanism to slow down T1D progression [117]. Upon arrival at pancreas, it is possible that Treg cells further undergo phenotypic and functional adaptations in the new microenvironment. By crossing Foxp3 scurfy mice with BDC2.5 mice, it is found that the absence of Treg does not affect T1D initiation but accelerates T1D progression. Additionally, the transcriptome profiling between PLN Treg and intra-islet Treg is different, suggesting that Treg cells primarily impinge on autoimmune diabetes by restraining destructive T cells inside the islets [118]. PLN-derived Treg cells are extremely potent and a mere 2,000 cells are capable of preventing diabetes development [119]. However, a study showed that miR-125a-5p is specifically hyper-expressed in Treg cells isolated from PLNs of donors with T1D. Upregulated miR-125a-5p is associated with reduced CCR2 level, which hinders the attraction of CCR2^+^ Treg cells by islet-derived CCL2 [120]. For the therapeutic purpose, butyrate administration induces colonic Treg cells and upregulates their surface expression of α4β7, CCR9, and GPR15, thereby directing their migration to PLNs and then pancreas [121]. The direct transfer of Treg cells suppresses the function of macrophages and inhibits effector T cell function in islets in a TGF-β-dependent manner, which lays the rational foundation of Treg-based T1D therapies [122].

## The intervention of T1D development by strategies targeting PLNs

From a PLN-centered view, T1D intervention strategy can be implemented by three major ways: cutting off the signal inputs into PLNs (reduce inflammatory β cell damage, enhance gut integrity and get rid of pathogenic viral infections), modulating the immune activation status of PLNs, and blocking the outputs of PLNs towards pancreatic islets.

Cell-based therapies: transfusion of tolerance-inducing cells is a feasible approach to restoring immune balance in PLNs. Apart from Treg cell transfer mentioned above, infused double-negative (DN) T cells preferentially home to PLNs, where they could suppress the function of CD4^+^ T cells and reverse new-onset T1D once applied in combination with anti-thymocyte serum (ATS) [123]. Similarly, intraperitoneal administration of IDO (indoleamine 2,3-dioxygenase) overexpressed fibroblasts manifested potency to attenuate islet inflammation by inducing Treg cells and decreasing autoreactive CD8^+^ T cells following migrating to local lymph nodes [124]. Moreover, DCs delivered by intravenous and/or intraperitoneal injection are predominantly drained to PLNs [125, 126]. Adoptively transferred IL-4 overexpressing BMDCs accumulate in PLNs, normalize the abnormal gene expression profile, and delay T1D progression [127]. 1,25-Dihydroxyvitamin D3 (1,25(OH)2D3) treatment induces tolerogenic dendritic cells (TolDCs) in both diabetes-prone NOD mice and diabetes-resistant C57BL/6 mice. Once the induced TolDCs are co-transferred with activated CD4^+^ T cells into NOD/SCID recipients, they dampen the proliferation of autoreactive T cells in PLNs [128].

Chemical-based therapies: small chemical compounds can be applied to T1D treatment and their action modes vary. One class of drugs works by disrupting the process of islet lymphocytic infiltration. Tellurium compounds, including AS101 and SAS, inhibit the activity of α4β7 integrin, thereby preventing autoreactive lymphocytes from migrating to the pancreas [129]. Tested in LEW.1AR1-IDDM spontaneous rat T1D model, S1P1 agonist FTY720 (fingolimod) promotes the retention of activated T cells in PLNs and hinders their islet infiltration [130]. By blocking the egress of lymphocytes and maintaining the integrity of peri-islet TLSs, FTY720 prevents diabetes development even at a time of significant insulitis in the spontaneous T1D model of NOD mice [24]. Alternative S1P1 receptor (S1P1R) modulator, ponesimod, inhibits the spreading of T cell responses and demonstrates a potential therapeutic effect when combined with an anti-CD3 antibody [131]. The other set of chemicals works by inducing tolerance in PLNs. Cytopiloyne from the plant *Bidens Pilosa* causes T cell apoptosis and elevates the Th2/Th1 ratio in PLNs [132]. Additionally, treatment with AHR ligand, 2,3,7,8-Tetrachlorodibenzo-p-dioxin (TCDD), expands Treg population and reduces pancreatic islet insulitis [133]. Administration of complete Freund's adjuvant (CFA) alone increases Treg percentage in PLNs and reverses new-onset T1D in 38% of NOD mice. The therapeutic effect is further boosted to 86% once it combines with the glucagon-like peptide-1 (GLP-1) analog exendin-4, which potently stimulates β cell replication [134]. Sulfatide reactive type II NKT cells (sulfatide/CD1d-tetramer^+^) are an anti-inflammatory subset differing from type I NKT cells. Administration of sulfatide C24:0 enlarges the type II NKT cell population, educates DCs to secrete more IL-10 and suppresses the activation of diabetogenic T cells [135]. Capsaicin, through binding to vanilloid receptor 1 (VR1), promotes anti-inflammatory macrophages in PLNs, which express IL-10 and PD-L1, and suppresses the activation of autoreactive T cells [136].

Vaccination-based therapies: vaccination has the advantage of inducing antigen-specific immune tolerance. Oral administration of recombinant insulin induces Treg cells in PLNs and shifts the Th1 response to Th2 by promoting the expression of IL-4 [137]. In addition, oral vaccination with live attenuated *Salmonella* that simultaneously delivers autoantigens and TGF-β induces tolerogenic DC throughout secondary lymphoid tissues and suppresses autoreactive T cell proliferation [138]. Moreover, delivery of microparticle formulation of RA (retinoic acid) plus TGF-β1 with the presence of islet autoantigen on the surface could induce tolerogenic DCs in PLNs, thereby preventing the progression of mid-stage autoimmunity to overt T1D [139]. Zymosan, the immunoregulatory adjuvant, bolsters the generation of tolerogenic DC subset via binding to TLR2 and Dectin1. Injection of NOD mice with β cell autoantigen and zymosan protects against T1D by facilitating the production of antigen-specific PLN Treg cells [140]. Moreover, intra-lymphatic administration of GAD-alum together with oral intake of vitamin D results in partial T1D remission in human patients, an effect ascribed to the elevated IL-10 secretion and reduced CD8^+^ T cell activation [141]. Autoantigen vaccination combined with nanotechnology and other immunoregulatory agents, therefore, represents a promising direction in the field of T1D treatment.

## Conclusions and perspectives

Finally, we conclude that PLNs serve as a pivotal hub linking various pathogenic inputs to islet β cell autoinflammatory damage. T1D intervention can be achieved by reducing pathogenic inputs/outputs and restoring the immune tolerant microenvironment of PLNs. Immunotherapies based on cell adoptive transfer, autoantigen vaccination, or chemical compounds should be combined with other therapeutic approaches, including probiotics that enhance gut integrity, β cell-protective agents (GLP-1) and those regulating vascular or lymphatic function. Regarding the dynamic and complex nature of T1D pathogenesis, the corresponding intervention strategy is better to be comprehensive.

To further extend the above-mentioned concept, T1D should be regarded as a systemic disease when organ/tissue communications are considered. Firstly, patients with T1D suffer from subclinical exocrine insufficiency and acinar atrophy although they are not as apparent as endocrine impairment [142]. A high degree of fibrosis is detected in the exocrine part while the precise mechanism is elusive, but suggested to be associated with global pancreatic inflammation, autoimmunity targeting the exocrine pancreas, vascular and neural anomalies, and the putative involvement of pancreatic stellate cells [143, 144]. Pancreatic exocrine function decreases in a majority of young at-risk children and precedes the onset of islet autoimmunity, as indicated by the measurement of exocrine biomarker, fecal elastase-1 (FE-1) [145]. Secondly, except for PLNs, spontaneous anti-insulin germinal centers (GC) are formed throughout lymphoid tissues [146]. Before the clinical onset of T1D, autoreactive T cells accumulate in the bone marrow and can respond to islet-derived antigen stimulation. Adoptively transferred bone marrow autoreactive T cells home back to PLNs and pancreas, which implies the complex systemic recycling of islet autoreactive T cells [147]. Thirdly, T1D is also subjected to neuronal regulation. Vagal nerves project to PLNs and pancreas and impact immune response. Pancreatic nerve electrical stimulation (PNES) retains T/B cells in PLNs and down-regulates the pro-inflammatory reaction to halt T1D progression in diabetic mice [148, 149]. Lastly, lymph node sharing accomplished by co-drainage of pancreas, liver and the upper small intestine (duodenum) has perplexed the regulation of pancreatic autoimmunity at the organismal level [150], and on the other way round, the involvement of PLNs in type 2 diabetes (T2D) associated hepatic/intestinal pathology should not be negated. Collectively, these lines of evidence bring about novel insights and remind a conceptual update on our current understanding of T1D pathogenesis.

## Data Availability

Not applicable.

## Abbreviations

T1D: Type 1 diabetes
LN: Lymph node
PLN: Pancreatic draining lymph node
DAMP: Damage associated molecular pattern
NOD: Non-obese diabetic
scRNA-seq: Single-cell RNA sequencing
Treg: Regulatory T cell
AICD: Activation-induced cell death
Teff: Exhaustion of effector T cells
TLO: Tertiary lymphoid organ
APC: Antigen-presenting cell
GC: Germinal center
DC: Dendritic cell
VEGFR3: Vascular endothelial growth factors receptor 3
MLDS: Multiple low dose streptozotocin
RRV: Rhesus monkey rotavirus
KRV: Kilham rat virus
pDC: Plasmacytoid DC
MHV-68: Murine gammaherpesvirus-68
DPP4: Dipeptidyl peptidase IV
NOD2: Nucleotide-binding oligomerization domain containing 2
MAdCAM-1: Mucosal addressin cell adhesion molecule 1
FDC: Follicular dendritic cell
HA: Hyaluronan
IAA: Insulin autoantibody
DEG: Differentially expressed gene
FRC: Fibroblastic reticular cell
NOR: Non-obese diabetes-resistant
LNSC: Lymph node stromal cell
DEAF1: Deformed epidermal autoregulatory factor 1
PTA: Peripheral tissue antigens
Eif4g3: Eukaryotic translation initiation factor 4 gamma 3
AIRE: Autoimmune regulator
DAP12: DNAX-activating protein of 12 k Da
IA-2: Insulinoma-associated antigen 2
ZnT8: Zinc transporter 8
MZB: Marginal zone B
STZ: Streptozotocin
MAdCAM-1: Mucosal addressin cell adhesion molecule-1
HEVs: High endothelial venules
IR: Insulin receptor
DN: Double-negative
IDO: Indoleamine 2,3-dioxygenase
1,25OH2D3: 1,25-Dihydroxyvitamin D3
TolDC: Tolerogenic dendritic cell
GLP-1: Glucagon-like peptide-1
VR1: Vanilloid receptor 1
RA: Retinoic acid
PNES: Pancreatic nerve electrical stimulation
